# Adherence to adrenaline autoinjector prescriptions in patients with anaphylaxis

**DOI:** 10.1186/s13601-019-0297-0

**Published:** 2019-11-08

**Authors:** Louise Parke, Annemarie Schaeffer Senders, Carsten Bindslev-Jensen, Annmarie Touborg Lassen, Athamaica Ruiz Oropeza, Susanne Halken, Sigurd Broesby-Olsen, Henrik Fomsgaard Kjær, Charlotte G. Mortz

**Affiliations:** 1 Department of Dermatology and Allergy Centre, Odense Research Centre for Anaphylaxis (ORCA), Odense University Hospital, Kløvervænget 15, 5000 Odense C, Denmark; 2 Department of Emergency Medicine, Odense University Hospital, 5000 Odense C, Denmark; 3 Hans Christian Andersen Children’s Hospital, Odense University Hospital, 5000 Odense C, Denmark

**Keywords:** Adherence, Adrenaline auto-injector, Anaphylaxis, Food allergy, Drug allergy, Mastocytosis, Prescription, Sampson’s severity score, Venom allergy

## Abstract

This study evaluates adherence to adrenaline autoinjector prescriptions in a cohort of well-characterized anaphylaxis patients. The overall retrieval rate was 76% with the highest rate in patients with severe anaphylaxis. Special attention is needed in patients with unknown elicitors and in young adults, comprising the largest proportion of non-adherent patients.

*Trial registration* No intervention performed. Retrospective data used with permission from the Danish Data Protection Agency and Regional Committees on Health Research Ethics

**To the editor**

Anaphylaxis is a severe, life-threatening systemic hypersensitivity reaction where first line treatment is intramuscular adrenaline [1]. Depending on the risk assessment long-term management of anaphylaxis includes prescription of an adrenaline autoinjector (AAI) which is often under-prescribed, not collected, not carried by the patient or not used in case of anaphylaxis [2, 3]. For AAIs the term adherence means the degree, to which the patient collects the prescription, carries the device and uses it correctly. In our study we aimed to determine adherence to AAI prescriptions, in a Danish cohort of well-characterized patients with anaphylaxis by comparing different elicitors (food, venom and unknown), severity of anaphylaxis, age, sex, and comorbidity. In this paper adherence is defined as collection of the AAI prescription at the pharmacy.

In a prospective study (APOTECA), 226 patients with suspected anaphylaxis were seen at the Emergency Department (ED), Odense University Hospital (OUH), during 1st of May 2013 through 30th of April 2014 [4]. In the study the suspected allergic reaction at the ED was classified according to the EAACI diagnostic criteria for anaphylaxis. By diagnostic work-up in accordance with the international guidelines at the Allergy Center (AC), OUH, anaphylaxis was confirmed in 124 of 226 patients [5]. Of the 124 patients one patient was seen in the ED outside the inclusion period, hence excluded from further analysis. Patients with drug anaphylaxis (n = 50) were excluded as they do normally not need an AAI. Furthermore, in 3 of the 13 patients with unknown elicitor an AAI was not prescribed and they were also excluded from the adherence study. Thus 70 patients were included in the present study. Children were defined as 0–17 years of age and adults ≥ 18 years. Severity of the reaction at the index date in the ED was evaluated according to Sampson’s severity score. All adults were screened for mastocytosis. Comorbidity such as asthma was registered.

Data on AAI retrievals prescribed from the ED or AC for patients with allergy to foods, venom and unknown elicitor was obtained from Odense Pharmacoepidemiological Database (OPED) [6] 1 year prior to and 1 year after the index date. OPED holds information on all reimbursed prescriptions from the Region of Southern Denmark (1.2 million inhabitants) since 1990. OPED is cross-linked to health-related registers via the Danish Civil Registration number. All drugs are registered after Anatomical Therapeutic Chemical (ATC) index. All AAIs (ATC: CO1CA24) require a prescription and includes the date of dispensation. The χ^2^-test was used for data comparison.

Table 1 shows the relationship between prescribed and retrieved AAIs in patients with confirmed anaphylaxis to food, venom and unknown elicitor, respectively. AAIs were purchased in 77% (20/26) of patients with food induced anaphylaxis, in 76% (26/34) of patients with anaphylaxis to venom and in 70% (7/10) of patients with anaphylaxis to unidentified elicitor. Significantly more patients with severe anaphylaxis retrieved an AAI (p < 0.02) compared to patients experiencing mild to moderate (Table 1).

**Table 1:** Prescribed and collected adrenaline autoinjectors according to elicitors, age, sex, severity, previous anaphylaxis and concomitant asthma or mastocytosis

	Food			Venom			Unknown elicitor			All elicitors
	Prescribed adrenaline autoinjector after anaphylaxis	Collected adrenaline autoinjector after anaphylaxis	%	Prescribed adrenaline autoinjector after anaphylaxis	Collected adrenaline autoinjector after anaphylaxis	%	Prescribed adrenaline autoinjector after anaphylaxis	Collected adrenaline autoinjector after anaphylaxis	%	% (proportion)
Total	26	20	76.9	34	26	76.5	10	7	70.0	75.7 (53/70)
Age										
Children (0–17 years)	13	11	84.6	0	–	–	4	4	100	88.2 (15/17)
Adults (≥ 18 years)	13	9	69.2	34	26	76.5	6	3	50.0	71.7 (38/53)
Sex										
Female	13	11	84.6	9	6	66.7	6	3	50.0	71.4 (20/28)
Male	13	9	69.2	25	20	80.0	4	4	100	78.6 (33/42)
Sampson’s severity score^a^										
Grade 1–3	2	2	100	7	3	42.9	2	0	0	45.5 (5/11)
Grade 4–5	24	18	75.0	27	23	85.2	8	7	87.5	81.4 (48/59)
Asthma	7	5	71.4	1	1	100	2	0	0	60.0 (6/10)
Mastocytosis^b^	0	–	–	4	4	100	1	0	0	80.0 (4/5)
Anaphylaxis > 1	14	9	64.3	13	9	69.2	6	4	66.7	66.7 (22/33)

All patients in the food (n = 26) and venom group (n = 34) had a prescription for an AAI. In 3 of the 13 patients with unknown elicitor an AAI was not prescribed due to primary elicitor suspicion on drug and they did not complete the diagnostic work-up. These 3 were excluded from the table. Number of prescriptions for AAI is unknown for the drug group and this group is not included in the table (n = 50)

^a^There was a significant difference between grade 1-3 and 4-5 anaphylaxis and retrieval of AAI (p < 0.02)

^b^The table includes 5 of 8 mastocytosis patients. Of the 3 not included 2 had anaphylaxis elicited by drug. Both patients picked up an AAI. One of the 3 had no AAI prescribed due to death before the evaluation was completed

Figure 1 illustrates percentage of collected AAIs in different age groups. Among young adults (18–35 years), only 45% (5/11) collected an AAI, whereas 81% (17/21) of patients older than 54 years retrieved their prescriptions (p < 0.04). Parents to children (< 18 years) collected an AAI more frequently than young adults (p < 0.01).

**Fig. 1:**
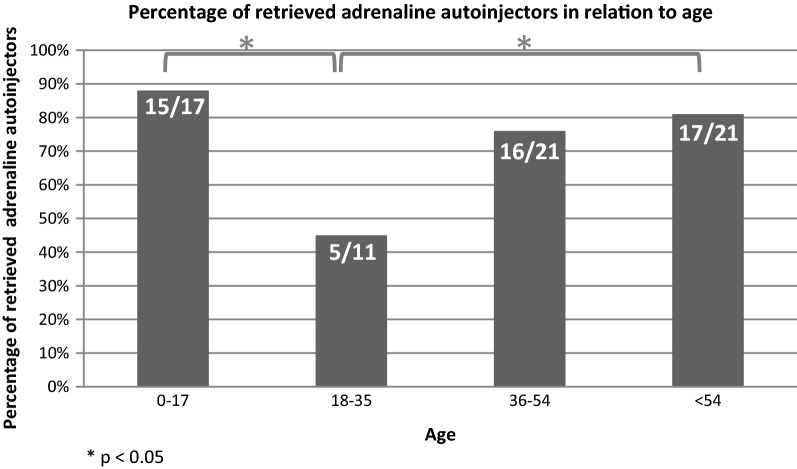
Percentage of retrieved adrenaline autoinjectors in relation to age

Patients collected their AAI within 1 month after the ED visit in 74% (39/53) of the cases. Three patients who did not retrieve an AAI within a year after the index date (ED visit) had collected an AAI the year before the index date. When including these patients the retrieval rate increased to 80% (56/70).

To our knowledge this is the first study to evaluate the level of adherence to AAI prescriptions in both children and adults with a verified diagnosis of anaphylaxis.

After diagnostic work-up in the AC the total retrieval rate of AAI was 76% (Table 1) which is in the upper end of a broad range of AAI retrieval rates (15–96%) hitherto reported [7–10]. Although partly reimbursed in Denmark, a reduced price for the AAI might improve compliance. Other likely explanations for non-adherence could be fear of needles; spontaneous recovery from previous anaphylaxis,—hence the assumption that adrenaline therefore would not be needed in future reactions either; reliance on oral antihistamines, glucocorticoids and/or inhaled bronchodilators; concerns about adrenaline’s adverse effects; and the patient’s own knowledge, risk assessment and concerns. Furthermore, the communication with and information given by the health care providers will affect adherence.

Despite thorough investigation (including co-factors and co-morbidities) at the AC, OUH, a culprit allergen could not be identified in all patients. Alarmingly, only 7/10 of these patients retrieved their prescriptions. Anaphylaxis with unknown elicitor is of concern due to the fact that the patient will not know which allergen to avoid. Thus, particular attention should be paid to possible overlooked causes for anaphylaxis, followed by a well-structured anaphylaxis management plan containing possible preventive strategies.

Severe reactions resulted in significantly higher retrieval rates (Table 1) for all elicitors compared to less severe reactions. Patients experiencing a severe anaphylaxis may fear and regard their disease as life-threatening inciting patients to retrieve their prescription.

Possibly, free AAIs would increase retrieval rates in our municipalities, potentially particularly in young adults with a low income. In Denmark only a part of the drug costs is reimbursed. In the present study, the least adherent group of patients was young adults between 18 and 35 years; only 45% retrieved their AAI. Our findings show by far the lowest AAI retrieval rate among recent studies for this age group [7, 8]. Their “critical age” may affect adherence and risk assessment due to emotional, physical and social changes including high-risk behavioral patterns regarding alcohol consumption and concomitant uncontrolled or partly controlled comorbidities such as asthma. This age group is at highest risk for fatal anaphylaxis to foods [11].

Previous studies have shown that many patients do not carry an AAI and do not know when and how to use adrenaline [3]. To improve adherence an anaphylaxis management plan should always be made together with the patient. Focus points in the management plan, in addition to continuing patient education in why, when and how to administer AAI should include the patient’s intraindividual, psychological perceptions [12, 13]: Questions such as the patient’s feeling of threat in relation to anaphylaxis, the patient’s opinion on the severity of his/her disease but also potential consequences of and negative aspects related to an allergic reaction need to be addressed. Furthermore, the health providers have to make sure that the patients have retrieved the AAI and bring it to the consultation and inform the patient always to carry it.

We conclude that even after diagnostic work-up in a highly specialized setting at the AC only 3 of 4 anaphylaxis patients retrieved their AAI prescription. This study thus highlights the need for patient education to ensure and strengthen adherence. A special focus should be on young adults and on adults where no elicitor could be identified. The strength of this study is that the patients are identified prospectively in the ED followed by a thorough diagnostic workup at a specialized Allergy Center to confirm the diagnosis and the elicitor. Furthermore, at our hospital no extraditions of AAIs are performed, all has to be retrieved at the pharmacy and a central system in Denmark allows us to follow the adherence to prescribed drugs. A limitation is the size of the study and that the results are only from a single center. Further studies on non-adherence will be needed in order to reach these groups of patients.

## Data Availability

Authors can confirm that all relevant data are included in the article and/or its additional information files.

## Abbreviations

AAI: adrenaline auto injector
AC: Allergy Center
ATC: Anatomical Therapeutic Chemical
ED: Emergency Department
EAACI: European Academy of Allergy and Clinical Immunology
OPED: Odense Pharmacoepidemiological Database
OUH: Odense University Hospital
